# South Beach Diet associated ketoacidosis: a case report

**DOI:** 10.1186/1752-1947-2-45

**Published:** 2008-02-11

**Authors:** Swapna Chalasani, Jacqueline Fischer

**Affiliations:** 1 Department of Internal Medicine, University of Illinois College of Medicine at Peoria, OSF Saint Francis Medical Centre, 530 NE Glen Oak Avenue, Peoria, IL 61637, USA

## Abstract

**Introduction:**

It has been previously unclear whether a "mild" degree of low carbohydrate or "starvation" ketonemia and acidosis induced by a low carbohydrate diet is clinically relevant to a patient.

**Case presentation:**

A 30-year-old Caucasian male on a low carbohydrate diet presented with nausea, vomiting and abdominal pain. The patient's bicarbonate level was 12 and he had hyperglycemia and ketonemia. He was felt to be in diabetic ketoacidosis and was started on intravenous insulin and isotonic saline infusions and responded well. Following cessation of insulin therapy, the patient remained normoglycemic for the remainder of his hospital stay. He later admitted to having been on the South Beach Diet, which is a low carbohydrate diet, for the three weeks prior to his presentation and during which time he had lost 16 pounds. On admission his BMI was 27.1. On presentation, the patient was felt to be in diabetic ketoacidosis but, interestingly, he was subsequently euglycemic without therapy. Following discharge, the patient discontinued the diet plan and he has remained asymptomatic and euglycemic over the following two years.

**Conclusion:**

The hyperglycemic ketoacidosis in this patient may have been caused by increased concentrations of free fatty acids in the absence of carbohydrate-induced inhibition of beta-oxidation of fatty acids and in the presence of an abnormally high ratio of glucagons to insulin. Given the present day popularity of low-carbohydrate diet plans, healthcare providers should be aware of the apparent association between such diets and symptomatic ketoacidosis. In a patient with ketoacidosis suspected to be secondary to a low carbohydrate diet, all other causes of high anion gap acidosis should be ruled out before attributing the acidosis to the low carbohydrate diet.

## Introduction

Low carbohydrate diets are nutritional programs that advocate restricted carbohydrate consumption based on research that ties consumption of certain carbohydrates with increased blood insulin levels, and overexposure to insulin with metabolic syndrome (the most recognized symptom of which is obesity). Under these dietary programs, foods high in digestible carbohydrates (sugars and starches) are limited or replaced with foods containing a higher percentage of proteins, fats and/or fiber. By contrast, if the diets are very low in starches and sugars (low-carbohydrate diets) the blood sugar level can fall so low that there is insufficient glucose to fuel the cells in the body. This state causes the pancreas to produce glucagon. Glucagon causes the conversion of stored glycogen to glucose and, once the glycogen stores are exhausted, causes the liver to synthesize ketones (ketosis) and glucose (gluconeogenesis) from fats and proteins. It has been previously unclear whether this "mild" degree of low carbohydrate or "starvation" ketonemia and acidosis induced by a low carbohydrate diet is clinically relevant to a patient.

## Case presentation

A 30-year-old Caucasian male without significant past medical history presented with a two day history of nausea, vomiting and diffuse abdominal pain. The patient denied use of any medications (prescription or nonprescription) or any illicit substances. He did admit to occasional ethanol ingestion stating that he consumed four alcoholic beverages (approximately 0.6 ounces ethanol each) the night prior to the onset of symptoms. The patient had a family history of diabetes mellitus type 2 on both the paternal and maternal side.

On presentation, the patient appeared in mild distress secondary to his stated abdominal pain. BMI on admission was 27.1 (weight 91 kilograms), vital signs were within normal limits, and the patient appeared euvolemic. Complete physical examination was normal including a normal abdominal examination. Initial laboratory studies revealed a high anion gap metabolic acidosis (arterial ph 7.34, arterial PCO_2 _23 mmHg, serum bicarbonate 12 mmol/L, serum anion gap 21) and hyperglycemia (serum glucose 267 mg/dL). The patient was found to have both ketonemia and ketonuria. Additional data, including a complete blood count, serum sodium, serum chloride, serum potassium, liver chemistries, lipid fractionation, serum lipase, serum amylase, plain chest radiography, and computed tomography of the abdomen and pelvis, were within normal limits. Serum osmolality, urine toxicology and lactic acid levels were not performed.

The patient was felt to be in diabetic ketoacidosis and was started on intravenous insulin and isotonic saline infusions to which he responded well with rapid resolution of the acidosis and abdominal pain within ten hours. Following cessation of the insulin therapy, the patient remained normoglycemic for the remainder of his hospital stay (24 hours). Hemoglobin A1C was 5.1% (4.4%–6.4%) and C peptide was 4.1 ng/mL (0.8–3.1 ng/mL).

The patient later admitted to having been on the South Beach Diet at the time of presentation, having adhered to a particularly strict (less than 20 grams carbohydrate daily) form of this low carbohydrate diet plan. The patient stated that he had eliminated virtually all forms of carbohydrate from his diet for the three weeks prior to his presentation and had lost 16 pounds (7.3 kg) over the same time period. Following discharge, the patient discontinued the low carbohydrate diet plan and he has remained asymptomatic and euglycemic over the following two years while maintaining a BMI of 27.

## Discussion

Here we present a case of hyperglycemic ketoacidosis associated with a low carbohydrate diet. The South Beach Diet is a popular diet plan which primarily relies on the restriction of dietary carbohydrates to achieve weight loss [1]. Our patient strictly adhered to 10 to 15 grams of carbohydrate per day for 3 weeks prior to presentation and lost 16 pounds. He was following the most stringent form of this diet, namely that being the form in which total carbohydrate consumption is limited to less than 20 grams daily. On presentation, our patient was felt to be in diabetic ketoacidosis but, interestingly, the patient was subsequently euglycemic without therapy and, even after two years of follow up, remained asymptomatic and euglycemic.

Low-carbohydrate, fat-rich meals stimulate glucagon secretion, lower insulin secretion, and increase insulin resistance [2,3]. Dietary and endogenous fat are catabolized to form ketone bodies as an energy source [4]. Plasma fatty acid concentrations can be two-fold higher during low-versus normo-carbohydrate diets in the postabsorptive period [5]. When the body has no free carbohydrates available, fat must be broken down into acetyl-CoA to generate energy. Acetyl-CoA is not being recycled through the citric acid cycle because the citric acid cycle intermediates (mainly oxaloacetate) have been depleted to feed the gluconeogenesis pathway, and the resulting accumulation of acetyl-CoA activates ketogenesis and this might have led to the ketoacidosis in our patient.

## Conclusion

Despite the widespread use of weight reducing low carbohydrate diets for many years now, few reports to date have highlighted their association with clinically relevant ketoacidosis [6,7]. This either means that it is a rare complication, or that it has, so far, not been recognized as a possible complication of a very strict low carbohydrate diet. The hyperglycemic ketoacidosis could easily, in the past, have simply been passed off as a complication of type 2 diabetes mellitus or metabolic syndrome (the low carbohydrate diet being viewed as an irrelevancy). It could also be that some people are applying the diet in an ever increasingly more fanatical way. A final possibility is that the syndrome is brought about by some, as yet unknown, trigger in persons on a very low carbohydrate diet.

Given the present day popularity of low-carbohydrate diet plans, healthcare providers should be aware of the apparent association between such diets and symptomatic ketoacidosis. In a patient with ketoacidosis suspected secondary to a low carbohydrate diet, all other causes of high anion gap acidosis should be ruled out before attributing the acidosis to the low carbohydrate diet. Although these laboratory tests were not performed in our patient, serum osmolal gap, lactic acid levels and salicylate levels, in addition to the tests which were performed in our patient, may be useful in ruling out other causes of acidosis.

## Competing interests

The author(s) declare that they have no competing interests.

## Authors' contributions

All authors have read and approved the final manuscript. SC: Involved in the conception of the report and literature review along with manuscript preparation, editing and submission. JF: Involved in the literature review, manuscript editing and manuscript review.

## Consent

Written informed consent was obtained from the patient for the publication of this study.
